# Integrated multi-omics analysis and experimental validation identify hub genes with diagnostic value in meningitis

**DOI:** 10.3389/fcimb.2026.1839548

**Published:** 2026-09-16

**Authors:** Anwaier Apizi, Aikebaier Tuerhong, Jian Li, Paiheriding Kamilijiang, Chun-Bo Yang, Zheng-Kai Wang, Rui-Feng Chai, Zhao-Xia Yu

**Affiliations:** Department of Intensive Care Unit, The First Affiliated Hospital of Xinjiang Medical University, Urumqi, China

**Keywords:** biomarker, epiregulin protein, HB-EGF protein, macrophage, meningitis

## Abstract

**Background:**

Meningitis remains a serious threat to human health; however, the molecular mechanisms underlying this condition, particularly those related to macrophage involvement, have not been fully elucidated. The aim of this study was to identify macrophage-associated hub genes in meningitis and to assess their diagnostic value and associations with immune cell infiltration.

**Methods:**

Multiple datasets from the Gene Expression Omnibus were analyzed to identify differentially expressed genes (DEGs). Immune cell infiltration was estimated using the CIBERSORT algorithm (Cell-type Identification by Estimating Relative Subsets of RNA Transcripts). Weighted Gene Co-expression Network Analysis (WGCNA) was conducted to identify key modules associated with classically activated (M1) macrophages. Hub genes were subsequently screened through integration of the protein–protein interaction (PPI) network with machine learning algorithms, including least absolute shrinkage and selection operator, support vector machine–recursive feature elimination, and random forest algorithms. Diagnostic performance was assessed using receiver operating characteristic curves and construction of a nomogram. Experimental validation was subsequently performed using reverse transcription quantitative polymerase chain reaction, western blotting, and flow cytometry in peripheral blood samples obtained from individuals with meningitis.

**Results:**

A total of 292 DEGs were identified, including 195 upregulated genes and 97 downregulated genes. Immune infiltration analysis demonstrated significantly increased proportions of M1 macrophages and activated dendritic cells in samples obtained from the meningitis group. Integrated analysis combining WGCNA, the PPI network, and machine learning methods identified *HBEGF* and *EREG* as hub genes. Both genes were significantly upregulated in individuals with meningitis, and the combined diagnostic area under the curve reached 0.922. Experimental validation confirmed elevated messenger RNA and protein expression levels of *HBEGF* and *EREG* in peripheral blood samples from individuals with meningitis. An increased proportion of M1 macrophages was observed.

**Conclusion:**

*HBEGF* and *EREG* were identified as key genes associated with M1 macrophage infiltration in meningitis. These genes may serve as potential diagnostic biomarkers for meningitis and provide clues for exploring future therapeutic targets.

## 1 Introduction

Meningitis is a severe inflammatory disorder of the meninges that poses a significant global threat to human health (Aksamit and Berkowitz, 2021). Based on etiological agents, meningitis can be bacterial, viral, fungal, or other less common forms (Poplin et al., 2020). Bacterial pathogens represent a common cause of meningitis, including *Streptococcus pneumoniae*, *Neisseria meningitidis*, and *Haemophilus influenzae*. Viral meningitis is commonly associated with infections caused by enterovirus, herpes simplex virus, and varicella-zoster virus. Fungal meningitis is relatively uncommon but may be caused by certain fungi, such as *Cryptococcus* and *Candida* (Al-Qahtani et al., 2022). According to data from the Global Burden of Disease Study, an estimated 236,000 deaths attributable to meningitis occurred worldwide in 2019, with approximately 2.51 million incident cases reported. The burden of meningitis remains particularly substantial in low- and middle-income countries, especially within the African meningitis belt, where both incidence and mortality rates are high (GBD 2019 Meningitis Antimicrobial Resistance Collaborators, 2023). In some countries within this region, the incidence of meningitis exceeds 100 cases per 100,000 population, and the mortality rate surpasses 20% (GBD 2019 Meningitis Antimicrobial Resistance Collaborators, 2023). Clinically, meningitis commonly presents with manifestations such as fever, headache, and vomiting and may also lead to neurological sequelae, including hearing loss, intellectual disability, and motor impairment, which substantially affect quality of life (Granton et al., 2024). Therefore, the development of effective therapeutic interventions to reduce the incidence and mortality of meningitis and mitigate the associated socioeconomic burden remains a critical priority.

Macrophages constitute an essential component of the immune system and play key roles in immune regulation, inflammatory responses, and in maintaining homeostasis under normal physiological conditions (Zhao et al., 2024). In tuberculous meningitis caused by *Mycobacterium tuberculosis*, macrophages recognize bacterial cell wall components through Toll-like receptors expressed on their surface, thereby initiating host immune responses (Ma et al., 2024). Previous investigations have demonstrated that macrophage dysfunction is closely associated with increased disease severity and poor prognosis in meningitis (Zhang and He, 2021). Pinho-Ribeiro et al. demonstrated that bacterial meningitis activates meningeal nociceptors to trigger calcitonin gene-related peptide (CGRP) secretion; CGRP further inhibits macrophage function and aggravates disease deterioration.

Ablation of receptor activity-modifying protein 1 expression in macrophages reduced the bacterial burden in the meninges and brain and increased macrophage expression of the chemokines *CXCL10*, *CCL3*, *CCL2*, and CGRP. These findings indicate that bacterial invasion triggers CGRP release, which subsequently suppresses meningeal macrophage activity and facilitates bacterial invasion (Houston, 2023).

Recent studies have identified a number of immune-related biomarkers for meningitis, such as S100B, GFAP and IL-6, which have shown good diagnostic performance in clinical practice (Zheng et al., 2018; DeKosky et al., 2021; Groeneveld et al., 2025). However, these biomarkers still have limitations in distinguishing different etiological types of meningitis and reflecting the underlying immune regulatory mechanisms. Therefore, this study aims to identify more specific and mechanism-related hub genes for meningitis. Currently, molecular-level investigations of meningitis remain limited, particularly with respect to the identification of hub genes and characterization of immune cell infiltration patterns. Identification of macrophage-associated hub genes in meningitis and characterization of immune cell infiltration patterns may contribute to the discovery of potential therapeutic targets and provide a theoretical foundation for the development of novel treatment strategies.

In the present study, bioinformatics approaches were applied to identify hub genes closely associated with macrophages in meningitis. Subsequently, peripheral blood samples obtained from individuals with meningitis were used to determine the expression levels of these hub genes and to analyze immune cell composition. The overall study workflow is presented in **Figure 1**. Furthermore, the associations between hub genes and immune cell infiltration were examined to elucidate potential mechanisms underlying macrophage functional regulation during meningitis progression. These findings may provide theoretical support for the diagnosis, treatment, and prognostic evaluation of meningitis and may contribute to the identification of potential therapeutic targets for improved disease management.

**Figure 1:**
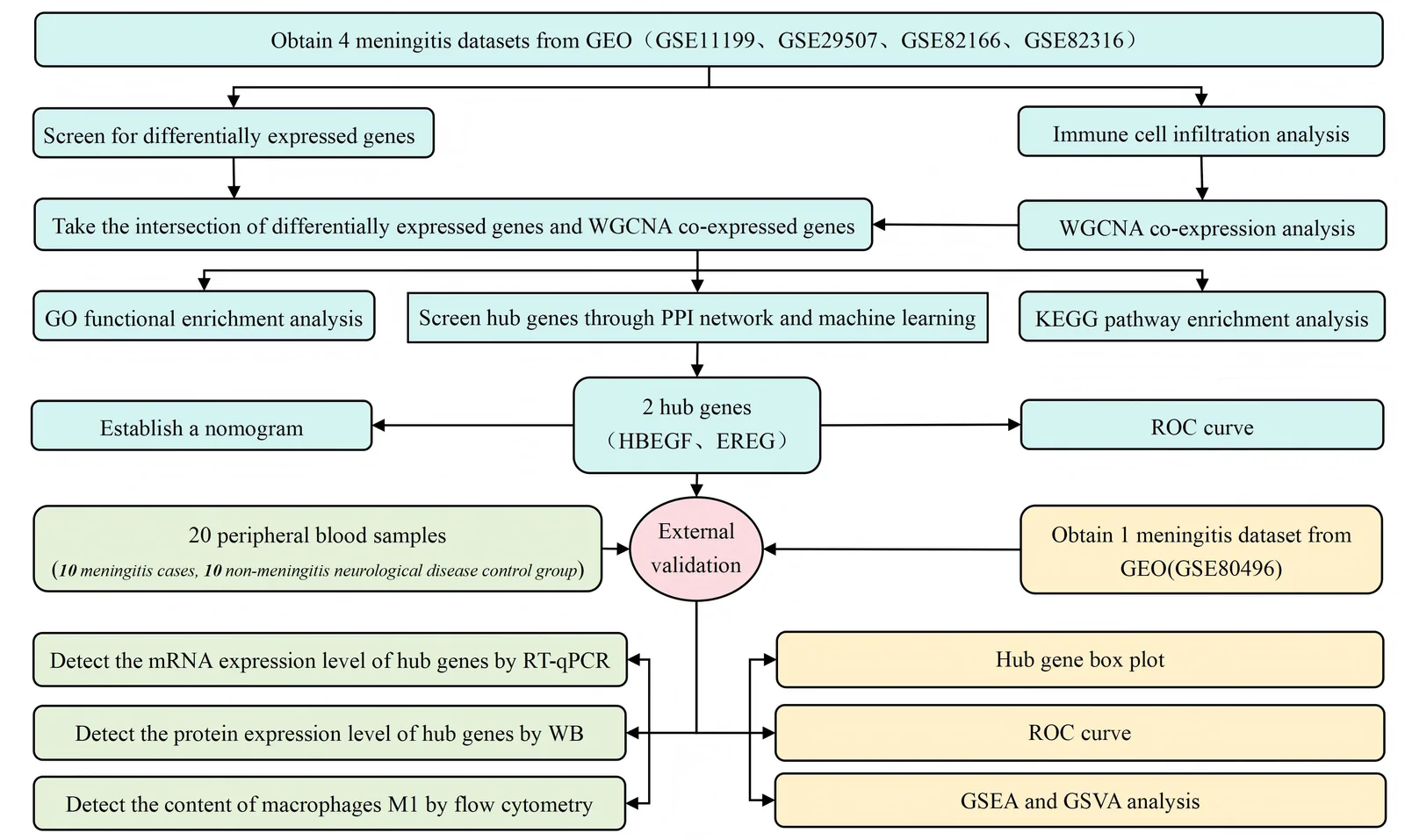
Overall workflow of bioinformatics analysis and experimental validation for meningitis hub gene screening.

## 2 Materials and methods

### 2.1 Data preprocessing and differential expression analysis

Raw gene expression datasets for meningitis and normal control samples (GSE11199, GSE29507, GSE82166, GSE82316, and GSE80496) were obtained from the Gene Expression Omnibus (GEO) database, as presented in **Supplementary Table 1**. The training set consisted of 15 normal controls and 18 meningitis samples, whereas the validation set consisted of 3 normal controls and 21 disease samples. Background correction, Robust Multi-array Average normalization, and probe annotation were conducted using the affy package in R (version 4.4.2). Probe identifiers were mapped to HUGO Gene Nomenclature Committee standard gene symbols, and the expression matrix was log_2_-transformed. Batch effects were corrected using the ComBat function in the sva package (version 3.54.0).

Principal component analysis (PCA) indicated that, after correction, the variance explained by PC1 increased to 85%, and batch-associated variation was substantially reduced. Following preprocessing of the GSE11199, GSE29507, GSE82166 and GSE82316 datasets, DEGs were identified using the limma package (version 3.62.1) with thresholds of |logFC| ≥ 1 and adjusted *P* value < 0.05. This threshold is the standard setting for transcriptome differential analysis, which balances the fold change of gene expression and statistical significance, and can effectively reduce false positive results. Volcano plots and heatmaps were generated using the pheatmap package (version 1.0.12), and clustering analysis was conducted using Euclidean distance and complete linkage methods.

### 2.2 Immune cell infiltration analysis and weighted gene co-expression network analysis

Immune cell infiltration was estimated using the Cell-type Identification by Estimating Relative Subsets of RNA Transcripts (CIBERSORT) algorithm to deconvolute gene expression profiles and estimate the relative abundance of 22 immune cell subtypes. The detailed parameter settings of CIBERSORT were as follows: the reference gene set was the LM22 gene set covering 22 immune cell subtypes, the number of permutations was 1000, quantile normalization was disabled, and absolute mode was not used. Bar plots to depict immune cell infiltration abundance and proportions across samples were generated using the ggplot2 package. Differences in immune cell proportions between meningitis and normal samples were compared using the Wilcoxon test (*p* < 0.05), and the results were visualized using stacked bar charts. Correlations among the 22 immune cell subtypes were analyzed using the corrplot package with default parameters, and statistical significance was assessed using 1,000 permutation tests (*p* < 0.05).

Weighted gene co-expression network analysis was performed using the Weighted Gene co-expression Network Analysis (WGCNA) implemented in the WGCNA package. The optimal soft-thresholding power was determined to be β = 6 (R² = 0.7) according to the scale-free topology criterion. Gene modules were identified through hierarchical clustering with a minimum module size > 30 genes. Key modules associated with macrophages in meningitis were screened based on module eigengene (ME) values and module membership (MM). Correlations between modules and immune cell infiltration levels were assessed using Pearson correlation analysis (*p* < 0.01). Overlap between DEGs and module genes was visualized using the VennDiagram package (version 1.7.3), and overlapping genes were identified.

### 2.3 Gene ontology and KEGG enrichment analysis of overlapping genes

Gene Ontology (GO) enrichment analysis includes three major categories: biological process (BP), cellular component (CC), and molecular function (MF) (Aleksander et al., 2023). Kyoto Encyclopedia of Genes and Genomes (KEGG) enrichment analysis is a bioinformatics approach based on the KEGG database, which integrates genomic, chemical, and systems-level functional data (Jin et al., 2023). Functional annotation of the overlapping genes was conducted using the clusterProfiler package (version 4.14.4), and the significance of KEGG pathway enrichment was assessed using the hypergeometric test (*p* < 0.05, q-value < 0.2). This threshold is the classic setting for enrichment analysis, which can ensure the reliability of enrichment results and avoid missing important disease-related pathways.

### 2.4 Identification of hub genes and construction of a nomogram

Protein–protein interaction (PPI) networks were obtained from the STRING database (version 12.0; confidence score > 0.4) and subsequently visualized and topologically analyzed using Cytoscape (version 3.10.3). The top 10 candidate hub genes were identified using the CytoHubba plugin based on the Maximal Clique Centrality algorithm. Three machine learning algorithms were applied to screen macrophage-related genes in meningitis: least absolute shrinkage and selection operator (LASSO) regression using the glmnet package (version 4.1-8; optimal λ selected through 10-fold cross-validation), support vector machine–recursive feature elimination (SVM-RFE) implemented in the e1071 package (version 1.7-16; radial basis function kernel), and random forest analysis using the randomForest package (version 4.7-1.2; 500 trees; variable importance assessed by mean decrease in accuracy). All models were assessed using 10-fold cross-validation for accuracy and area under the curve (AUC). Hub genes were determined through integration of results from Venn diagram and circos plot analyses.

Receiver operating characteristic (ROC) curves were generated using the pROC package (version 1.18.5), and an AUC ≥ 0.7 was considered indicative of acceptable discriminative ability. Risk scores were calculated according to a predefined risk score formula, and a nomogram was constructed using the rms package (version 7.0-0). Predictive agreement between the nomogram and observed outcomes was assessed using a calibration curve. The GSE80496 dataset was used to validate the hub genes using a *t*-test (*p* < 0.05), and hub genes that were differentially expressed in the validation dataset were selected for subsequent analyses.

To reduce the risk of overfitting in the small high-dimensional dataset, all machine learning models adopted 10-fold cross-validation to select the optimal parameters. The optimal λ value of LASSO regression was selected through 10-fold cross-validation, which effectively reduced the risk of overfitting. Three different machine learning algorithms (LASSO, SVM-RFE, random forest) were used for cross-validation, and only the genes screened out in all three algorithms were identified as hub genes, which avoided the bias of a single algorithm. The calibration curve of the nomogram showed a good consistency between the predicted probability and the actual observed value, indicating no obvious overfitting in the model. The external validation dataset GSE80496 further verified the diagnostic performance of the hub genes, which proved the generalization ability of the model.

### 2.5 Gene set variation analysis and gene set enrichment analysis

Gene sets were obtained from the Molecular Signatures Database (MSigDB). Gene Set Variation Analysis (GSVA) was conducted using the GSVA package (version 2.0.5) to quantify pathway activity. Differentially enriched pathways were identified using the limma package with thresholds of |logFC| > 0.5 and *p* < 0.05. Gene Set Enrichment Analysis (GSEA) was conducted using the clusterProfiler package based on MSigDB gene sets (version 7.5.1), with 1,000 permutations and *p* < 0.05.

### 2.6 Clinical sample collection and validation

Peripheral blood samples were collected from 10 individuals with meningitis and 10 individuals from the non-meningitis neurological disease control group at The First Affiliated Hospital of Xinjiang Medical University for validation of gene expression profiles using reverse transcription quantitative polymerase chain reaction (RT-qPCR), western blotting (WB), and flow cytometry. Written informed consent was obtained from all participants, and the study was approved by the hospital ethics committee. Two tubes of EDTA-anticoagulated peripheral blood (3 mL per tube) were collected from each participant. One tube was mixed with 6 mL of TRIzol reagent (Leagene, Beijing, China) to ensure complete cellular lysis, followed by centrifugation and storage at −80 °C until analysis. The second tube was transported to the laboratory within 2 hours in a cold transport container maintained at 4 °C for immediate processing. Baseline clinical characteristics (age, sex, primary diagnosis, comorbidities and antibiotic exposure prior to sampling) of all enrolled participants are summarized in **Supplementary Table 4**.

### 2.7 RNA isolation and RT-qPCR

Total RNA was extracted using TRIzol reagent (Leagene, Beijing, China) according to the instructions provided by the manufacturer. Reverse transcription was performed using a Reverse Transcription Kit (Foregene, Chengdu, China) containing random primers and oligo(dT) primers to generate complementary DNA (cDNA). Quantitative PCR was subsequently performed on a Light-Cycler 480 Instrument II (Roche) using qPCR Premix (Foregene, Chengdu, China). Thermal cycling conditions were as follows: initial denaturation step at 95 °C for 10 minutes, followed by 40 amplification cycles (95 °C for 15 seconds and 60 °C for 10 seconds), and a final extension step at 72 °C for 10 seconds. Relative expression levels of target genes were calculated using the 2^−ΔΔCt^ method. The primer sequences used in this study are provided in **Supplementary Table 2**.

### 2.8 Western blotting

WB was conducted to validate the protein expression levels of the model-related genes. Samples were lysed in RIPA buffer (Thermo Fisher Scientific), and protein concentrations were determined using the Pierce BCA Protein Assay Kit (Thermo Fisher Scientific). Equal amounts of protein (20 μg) were loaded onto gels for electrophoresis and subsequently transferred onto PVDF membranes (Millipore). After blocking with 5% non-fat milk, membranes were incubated overnight at 4 °C with primary antibodies against *HBEGF* and *EREG* (both from ABclonal Technology, Wuhan, China) at a dilution of 1:1,000. Horseradish peroxidase-conjugated secondary antibody (Cell Signaling Technology) was then applied and incubated at room temperature for 40 minutes. Target proteins were visualized using an enhanced chemiluminescence kit (Biosharp, Hefei, China). During image acquisition and densitometric analysis, two samples (one from each group) were excluded due to relatively low ACTB internal reference signal and poor band quality, leaving 9 samples per group for WB quantification.

### 2.9 Flow cytometry for detection of M1 macrophages in peripheral blood

Peripheral blood mononuclear cells (PBMCs) were isolated from peripheral blood by density gradient centrifugation using Lymphocyte Separation Medium (Beyotime Biotechnology, Shanghai, China). Diluted blood samples were layered onto the separation medium and centrifuged at 400 × g. The buffy coat layer was carefully collected, washed twice with pre-chilled phosphate-buffered saline (PBS) containing 2% fetal bovine serum, and resuspended for cell counting. An appropriate number of PBMCs were sequentially subjected to surface marker staining, fixation and permeabilization, and intracellular staining under light-protected conditions at 4 °C.

The antibody panel included anti-CD86 (ABclonal Technology, Wuhan, China) and anti-iNOS (Abcam, Cambridge, UK). 7-aminoactinomycin D (7-AAD; Beyotime Biotechnology) was added to exclude non-viable cells. Samples were analyzed using a BD LSRFortessa flow cytometer, which was calibrated with standard microspheres prior to acquisition. Fluorescence compensation was adjusted using single-stained controls. Gating was performed based on forward scatter and side scatter parameters to exclude cellular debris, and a minimum of 1 × 10^5^ viable events were acquired for each sample. Data analysis and gating were conducted using FlowJo software (version 10.10), and the relative proportion of M1 macrophages within the PBMC population was determined.

### 2.10 Statistical analysis

Statistical analyses, including quality control and preprocessing of gene expression data, were performed using the limma package in the R platform. The Wilcoxon rank-sum test was applied to compare the proportions of 22 immune cell types between samples obtained from individuals with meningitis and normal control samples, with *p* < 0.05 considered statistically significant. The limma package was used to identify genes with a false discovery rate < 0.05 and a |log_2_ fold change| ≥ 1 for WGCNA. Correlations between gene modules and immune cell abundance were assessed using Pearson correlation analysis (*p* < 0.01). Differences in hub gene expression between the two groups were validated using the Student’s *t*-test, with *p* < 0.05 considered statistically significant.

## 3 Results

### 3.1 Screening of DEGs in meningitis

Principal component analysis (PCA) indicated that, after correction, the variance explained by PC1 increased to 85%, and batch-associated variation were substantially reduced, and samples were clearly separated according to disease state, indicating that batch effect correction was effective and disease-related biological signals were well retained.

PCA demonstrated distinct global gene expression patterns between samples obtained from individuals with meningitis and normal control samples (**Figures 2A, B**). Differential expression analysis was conducted using thresholds of adjusted *p* < 0.05 and |logFC| ≥ 1. The resulting volcano plot (**Figure 2C**) identified a total of 292 DEGs, including 97 downregulated genes and 195 upregulated genes. A heatmap was generated to visualize the top 50 significantly upregulated and top 50 downregulated genes (**Figure 2D**).

**Figure 2:**
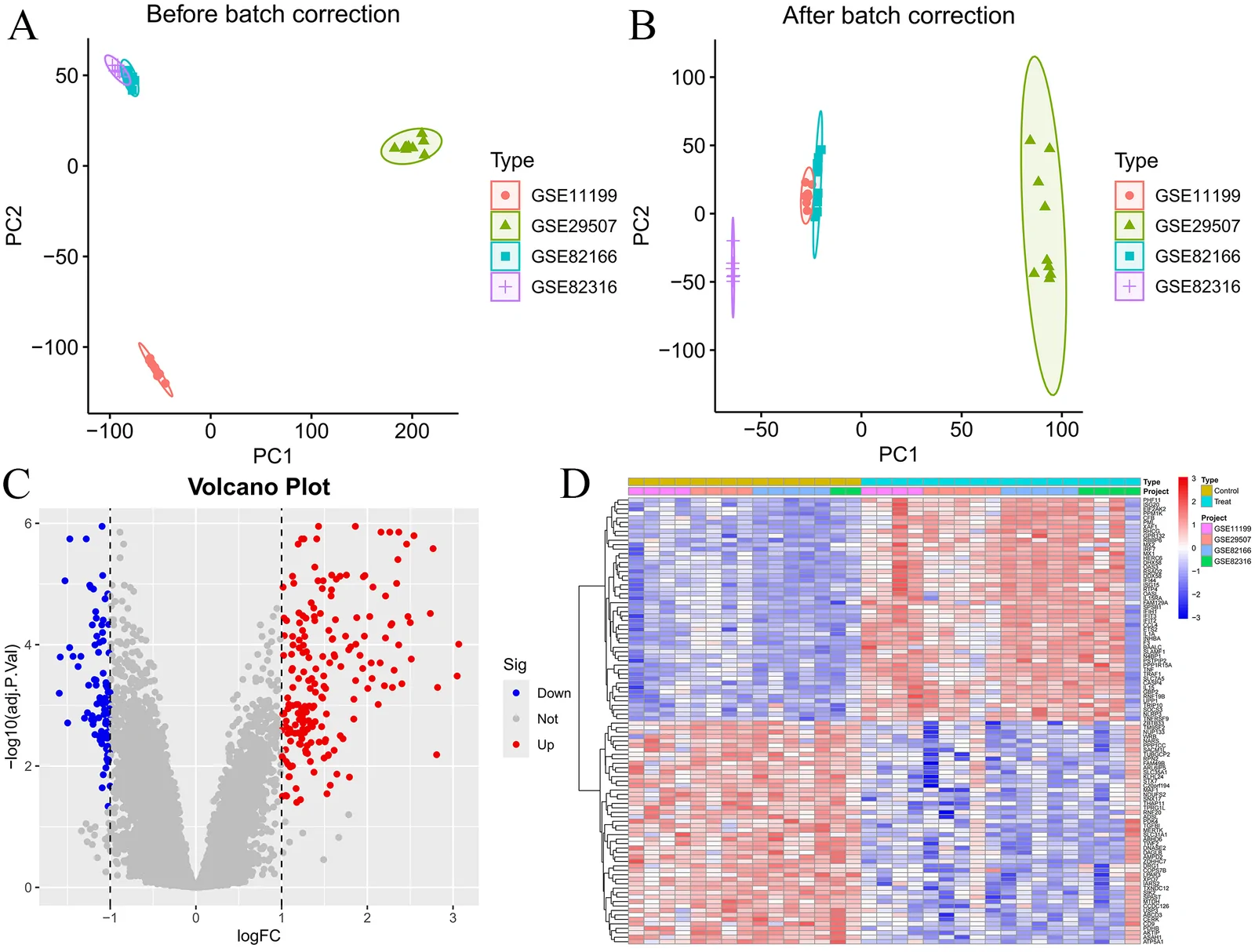
Data preprocessing, batch effect correction and differentially expressed gene (DEG) identification. **(A)** PCA of four original meningitis datasets prior to batch-effect correction. **(B)** PCA of the integrated meningitis dataset after batch-effect correction. **(C)** Volcano plot of DEGs in the integrated meningitis dataset. Upregulated genes are indicated by red dots, whereas downregulated genes are indicated by blue dots. **(D)** Heatmap presenting the top 50 downregulated and top 50 upregulated DEGs in the integrated meningitis dataset. PCA, Principal component analysis; DEGs, differentially expressed genes.

### 3.2 Immune cell infiltration analysis

The relative proportions of 22 immune cell types in each sample were initially visualized using a stacked bar plot (**Figure 3A**). Violin plots (**Figure 3B**) were subsequently generated to compare the proportions between the meningitis and control groups. The results indicated that the proportions of monocytes (*p* < 0.001), M0 macrophages (*p* < 0.001), M1 macrophages (*p* < 0.001), M2 macrophages (*p* < 0.01), resting dendritic cells (*p* < 0.05), activated dendritic cells (*p* < 0.05), activated mast cells (*p* < 0.001), and neutrophils (*p* < 0.01) differed significantly between the two groups. Increased proportions of M1 macrophages, activated mast cells, and activated dendritic cells were observed in the meningitis group. A correlation heatmap illustrating interactions among immune cell subtypes (**Figure 3C**) indicated a negative correlation between resting natural killer (NK) cells and activated NK cells (coefficient = −0.87). In contrast, a strong positive correlation was observed between gamma-delta T cells and follicular helper T cells (coefficient = 0.97). A moderate positive correlation was identified between M1 macrophages and gamma-delta T cells (coefficient = 0.52).

**Figure 3:**
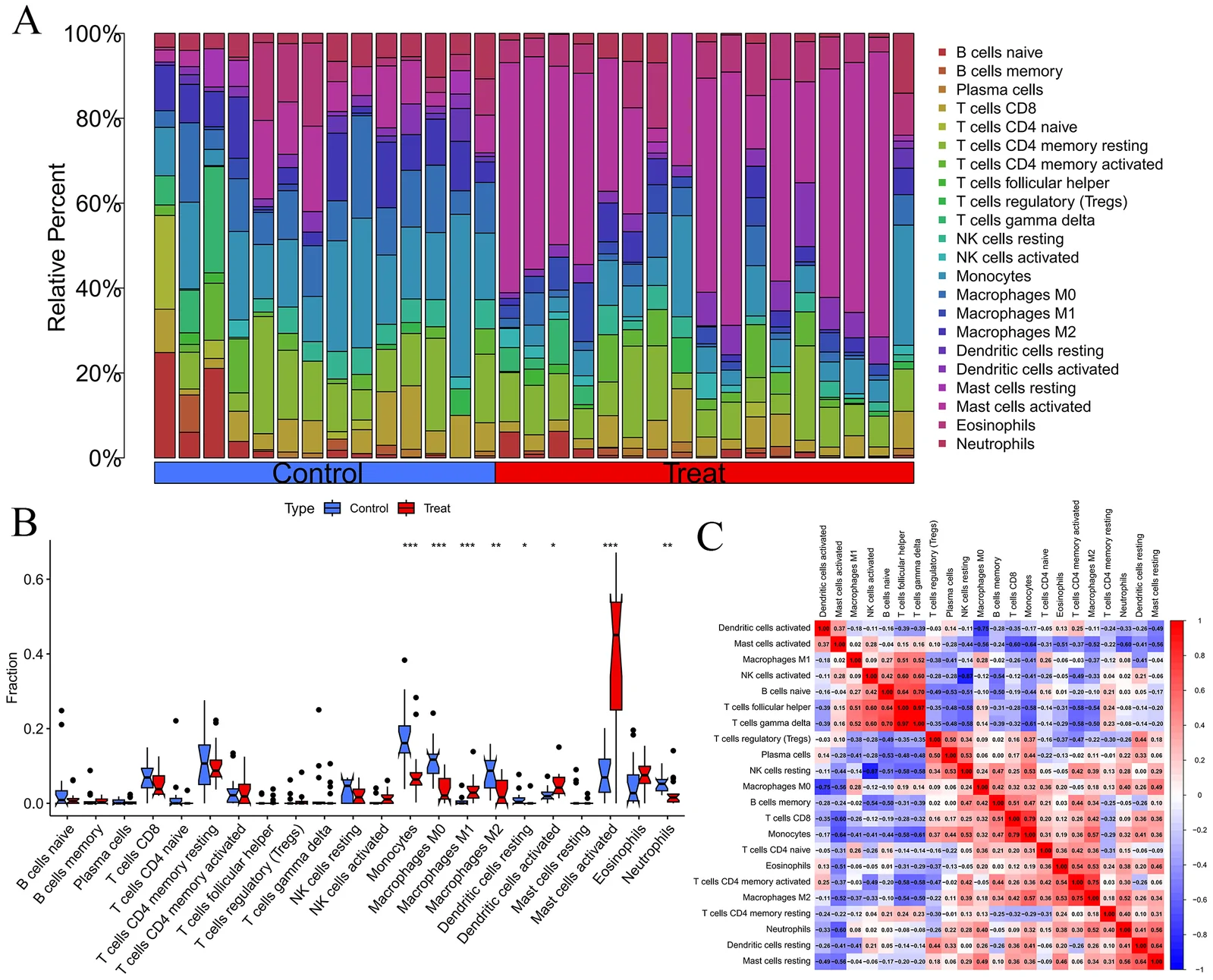
Estimation of immune cell infiltration landscapes via CIBERSORT algorithm. **(A)** Stacked bar chart depicting immune cell proportions in the meningitis and control groups. **(B)** Violin plot comparing 22 immune cell types between the meningitis and control groups. *p < 0.05; **p < 0.01; ***p < 0.001; ****p < 0.0001. **(C)** Heatmap depicting correlations among 22 immune cell types at *P* < 0.05.

### 3.3 Weighted gene co-expression network analysis

To further identify key genes associated with meningitis, WGCNA was conducted to identify gene modules significantly associated with meningitis. Based on the scale-free topology criterion, a soft-thresholding power of 6 was selected (**Figure 4A**). 13 modules were generated and visualized by a clustering dendrogram using this threshold (**Figure 4B**). Correlation analysis between immune cell infiltration and gene modules (**Figure 4C**) indicated that the salmon module exhibited the strongest correlation with M1 macrophages (coefficient = 0.54, *p* = 0.02) and was therefore selected for further analysis. A scatter plot (**Figure 4D**) further demonstrated a positive correlation between gene significance (GS) for M1 macrophages and MM (coefficient = 0.49, *p* = 3e−07). Overlapping genes were subsequently identified by intersecting the salmon module genes with the identified DEGs, and the results were visualized using a Venn diagram.

**Figure 4:**
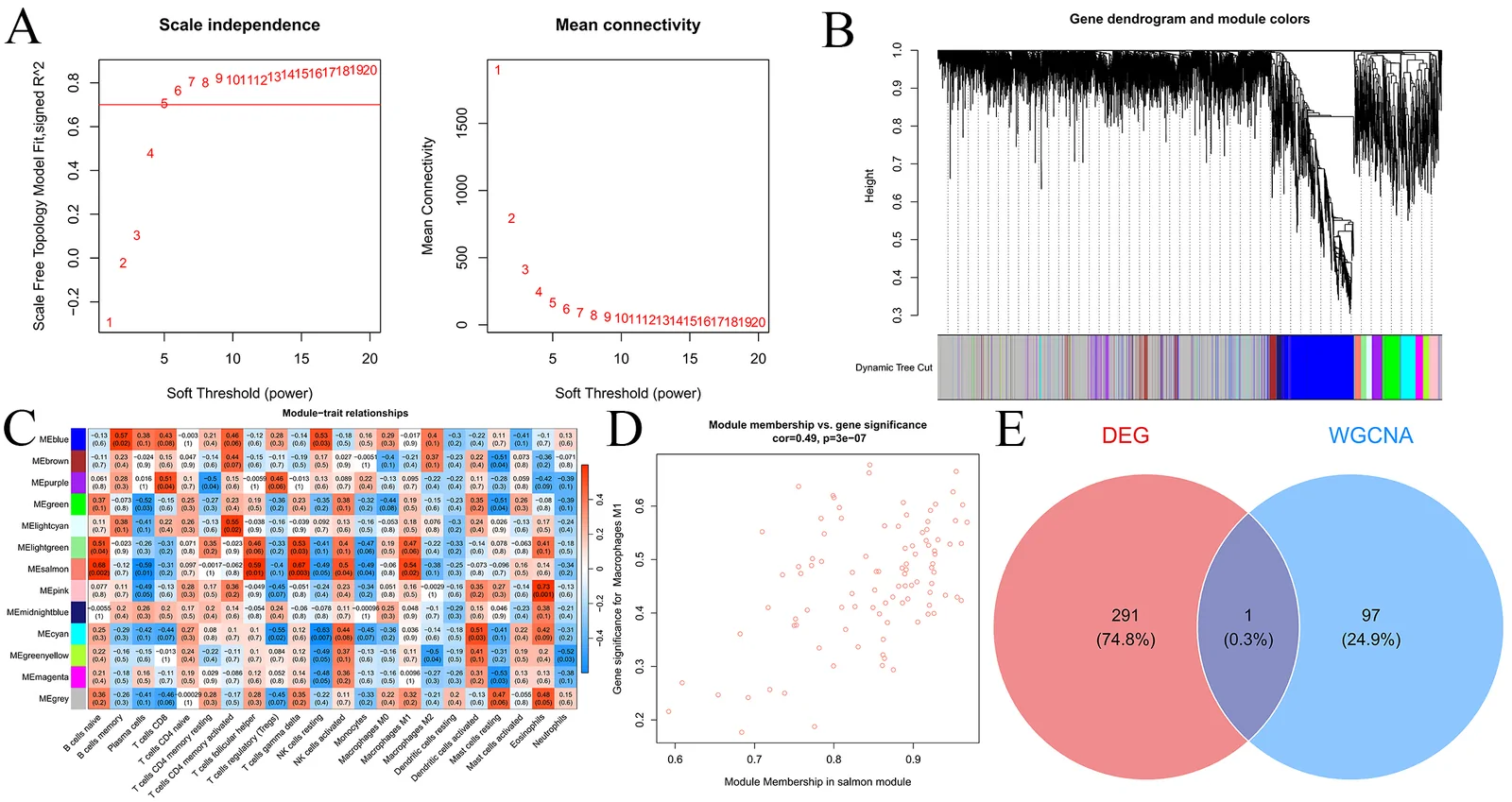
Weighted gene co-expression network analysis (WGCNA) and intersection of DEGs and module genes associated with M1 macrophages. **(A)** Determination of the scale-free topology fit index; scale-free R² = 0.7, with a soft-thresholding power = 6. **(B)** Clustering dendrogram of genes identifying 13 modules. **(C)** Correlation heatmap between different gene modules and immune cells. **(D)** Correlation between module membership (MM) and gene significance (GS) for M1 macrophages within the salmon module. **(E)** Venn diagram depicting intersecting genes between the salmon module and differentially expressed genes (DEGs).

### 3.4 GO and KEGG enrichment analysis

GO analysis was conducted to examine the biological functions of the differentially expressed intersection genes, and bar plots (**Figure 5A**) and bubble plots (**Figure 5B**) were generated. The results indicated that the DEGs were primarily associated with BP, MF, and CC categories. Within the BP category, genes were predominantly enriched in humoral immune response-related processes. In the CC category, enrichment was mainly observed on the external side of the plasma membrane. Within the MF category, enrichment was primarily associated with phospholipase binding. KEGG pathway analysis was conducted to examine the signaling pathways associated with the differentially expressed intersection genes, and bar plots (**Figure 5C**) and bubble plots (**Figure 5D**) were generated. The results indicated that the DEGs were mainly enriched in the ErbB signaling pathway, colorectal cancer, MAPK signaling pathway, and PI3K-Akt signaling pathway.

**Figure 5:**
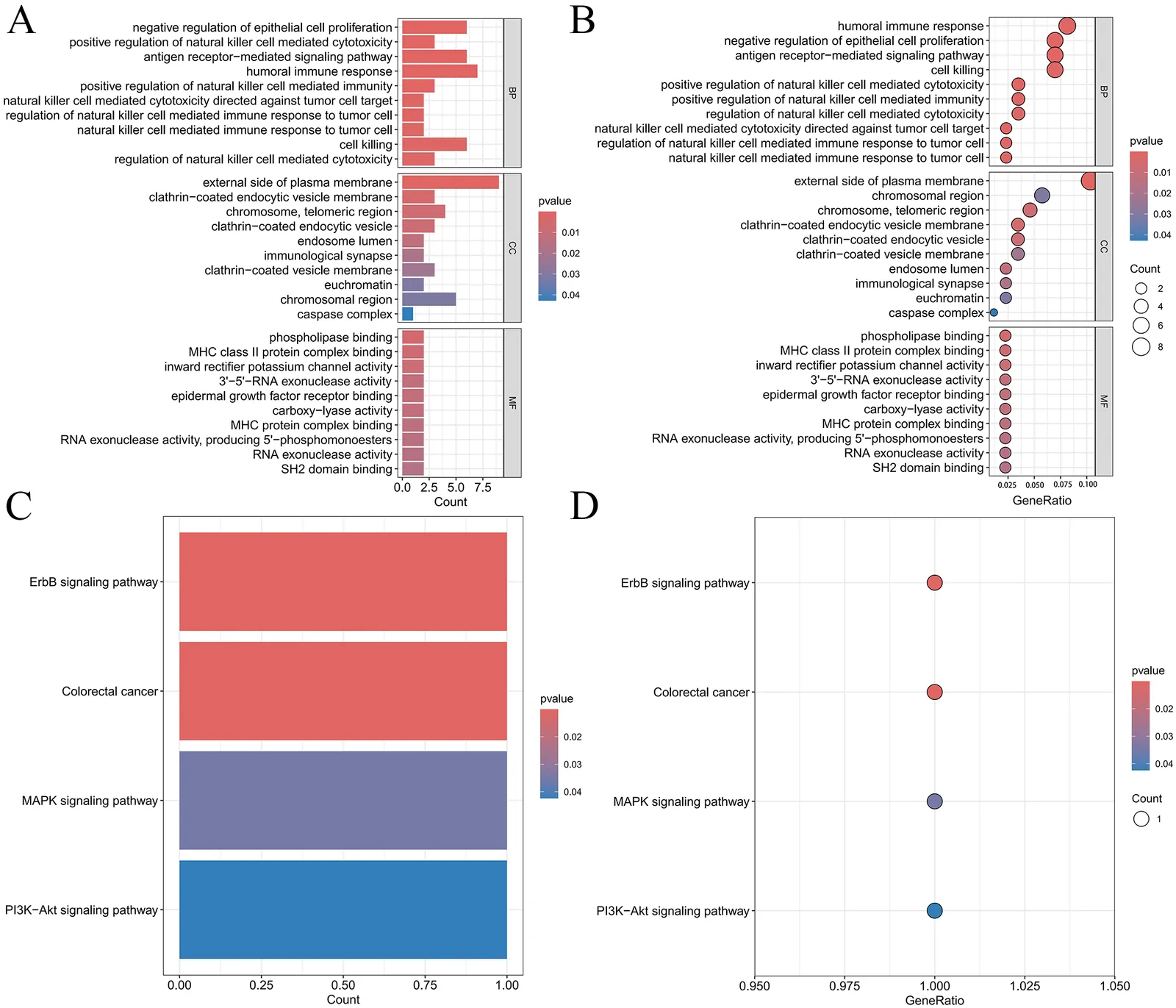
GO functional enrichment and KEGG pathway enrichment analysis of intersecting genes between DEGs and salmon module genes. **(A)** Bar plot of GO enrichment analysis. **(B)** Bubble plot of GO enrichment analysis. **(C)** Bar plot of KEGG enrichment analysis. **(D)** Bubble plot of KEGG enrichment analysis.

### 3.5 Identification of hub genes

A PPI network was constructed using the STRING database to explore interactions among the screened DEGs. The network was visualized, and the top 10 hub genes associated with meningitis were identified (**Figure 6A**). These 10 hub genes were subsequently subjected to LASSO regression analysis (**Figure 6B**), SVM-RFE analysis (**Figure 6C**), and random forest analysis (**Figures 6D, E**). Integration of the results using a Venn diagram (**Figure 6F**) identified *HBEGF* and *EREG* as two hub genes associated with M1 macrophages in meningitis. The relationships between these genes were further visualized using a circos plot (**Figure 6G**).

**Figure 6:**
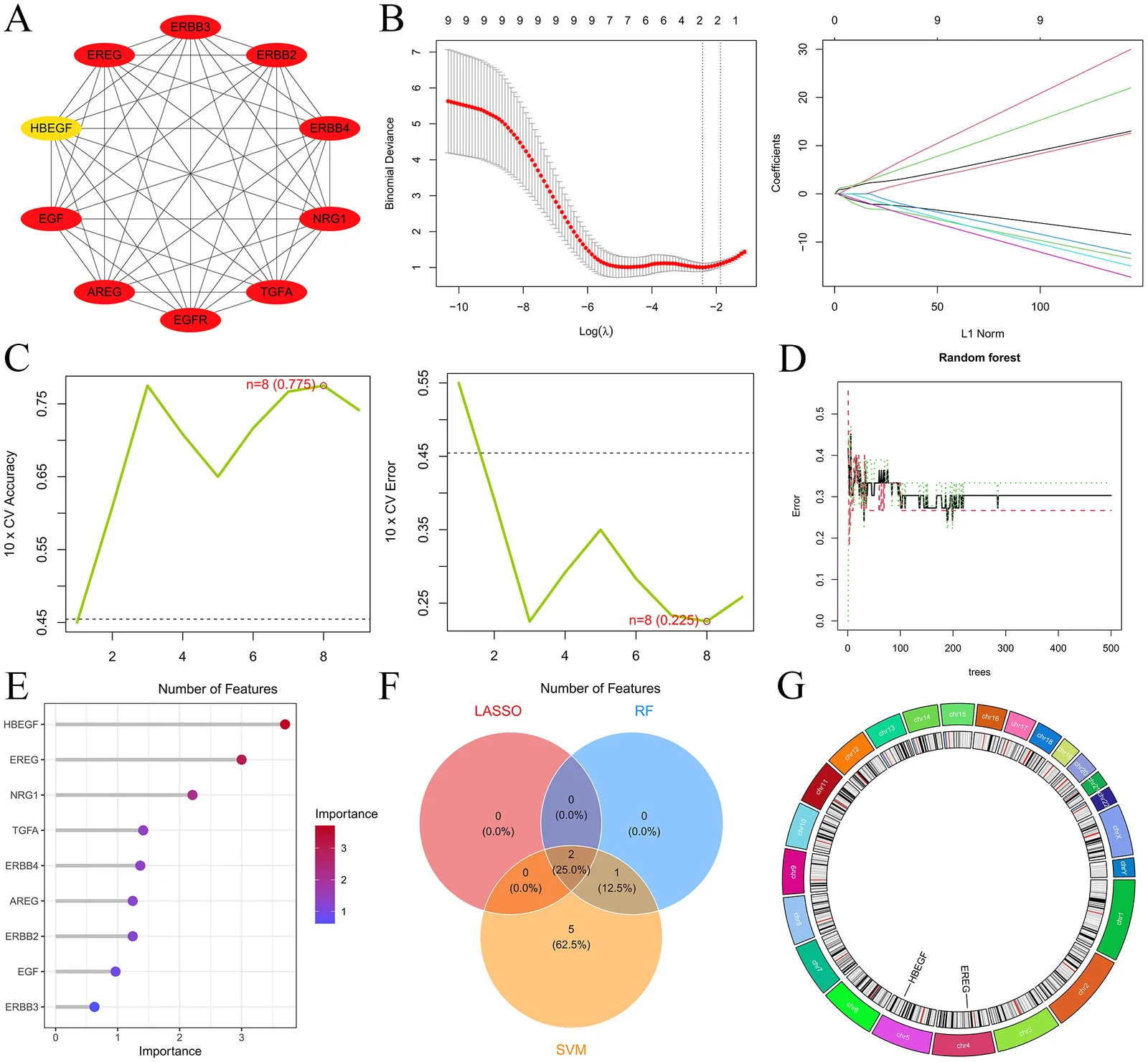
PPI protein interaction network and three machine learning algorithms for hub gene screening. **(A)** PPI network illustrating the top 10 hub genes. **(B)** Least absolute shrinkage and selection operator (LASSO) regression analysis. **(C)** Support vector machine–recursive feature elimination (SVM-RFE) analysis. **(D)** Random forest plot. **(E)** Lollipop plot presenting hub gene importance in the random forest algorithm. **(F)** Venn diagram illustrating the overlap among the three algorithms. **(G)** Circos plot depicting the two hub genes.

### 3.6 Construction and validation of the nomogram

The expression levels of the two hub genes across four datasets were compared using *t*-tests, and the results were visualized using box plots (**Figure 7A**). The results indicated that *HBEGF* (*p* < 0.05) and *EREG* (*p* < 0.001) exhibited statistically significant differences in expression between the meningitis and control groups, with both genes presenting higher expression in the meningitis group. ROC curves were generated to assess the predictive performance of individual hub genes (**Figure 7B**) and the combined predictive model (**Figure 7C**). *HBEGF* (AUC = 0.759) and *EREG* (AUC = 0.893) both demonstrated good predictive ability, whereas the combined model presented excellent predictive capacity for meningitis risk (AUC = 0.922, 95% CI: 0.811–0.989). Based on the risk score formula: Risk Score = 1.57 × *HBEGF* Exp + 2.83 × *EREG* Exp, a nomogram was constructed to predict meningitis risk using the two hub genes (**Figure 7D**). A calibration curve (**Figure 7E**) was generated to further assess the predictive performance of the model.

**Figure 7:**
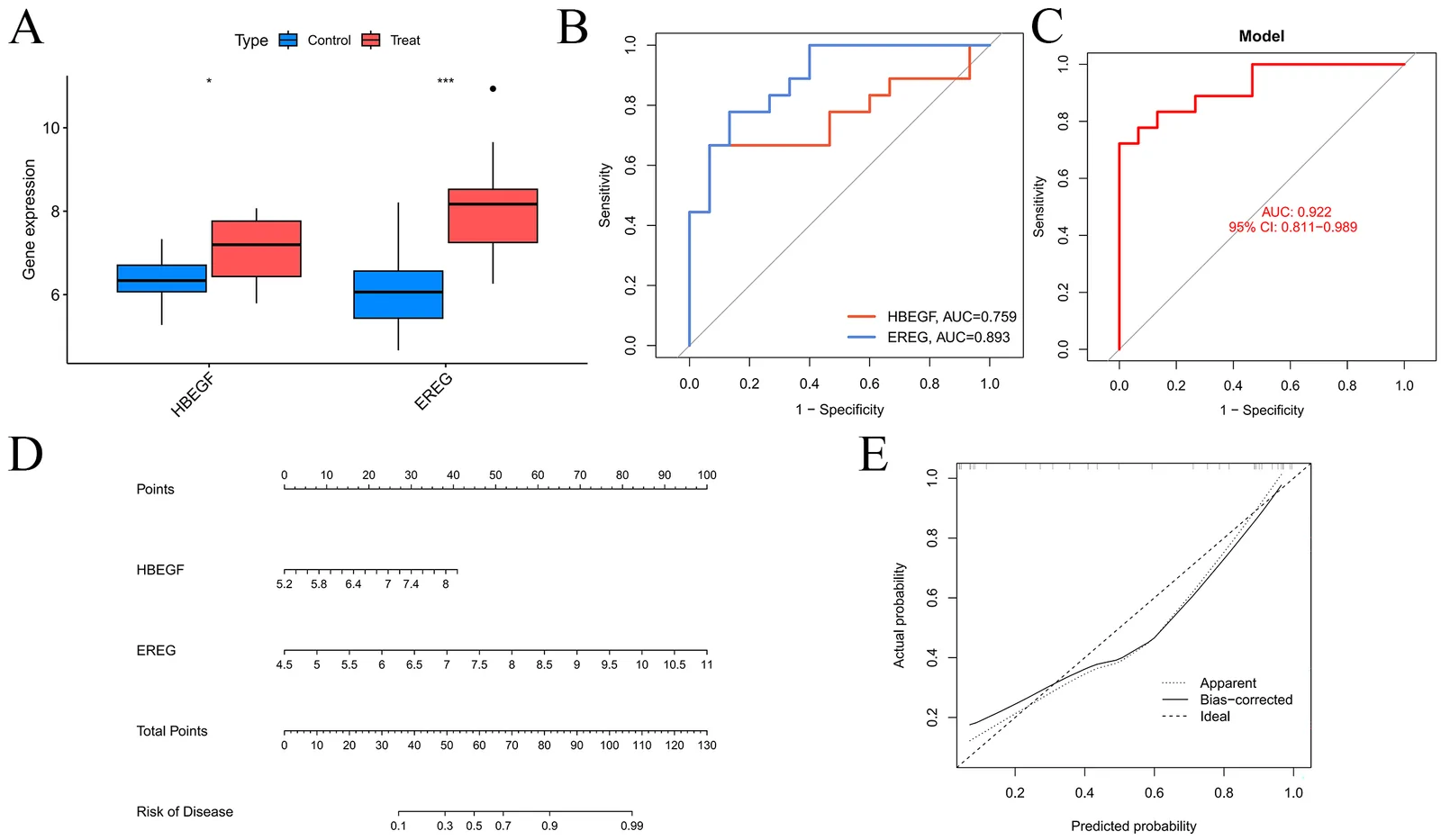
Diagnostic performance of HBEGF and EREG in the training dataset. **(A)** Box plots presenting the expression levels of the two hub genes. *p < 0.05; **p < 0.01; ***p < 0.001. **(B)** Receiver operating characteristic (ROC) curves of the two hub genes. **(C)** Area under the curve (AUC), the logistic regression model for predicting meningitis risk. **(D)** Nomogram constructed based on the two hub genes. **(E)** Calibration curve illustrating the diagnostic performance of the nomogram model.

### 3.7 External validation and enrichment analysis

Following construction of the nomogram, the expression levels of the two hub genes were further validated using the GSE80496 dataset. Box plots (**Figure 8A**) indicated that both *HBEGF* and *EREG* were highly expressed in the meningitis group; however, only *HBEGF* presented a statistically significant difference between the meningitis and control groups (*p* < 0.01). ROC curve analysis was conducted to assess the sensitivity and specificity of *HBEGF* (**Figure 8B**), and the results demonstrated excellent predictive value for meningitis risk (AUC = 0.968). Samples in the training dataset were stratified into high- and low-expression groups according to the median proportion of M1 macrophages. The external validation dataset GSE80496 included 3 control samples and 21 meningitis samples. Although the number of control samples was small, t-test results showed that HBEGF had a significant expression difference between the two groups (p < 0.01), and the AUC of the ROC curve reached 0.968, which still showed good diagnostic performance.

**Figure 8:**
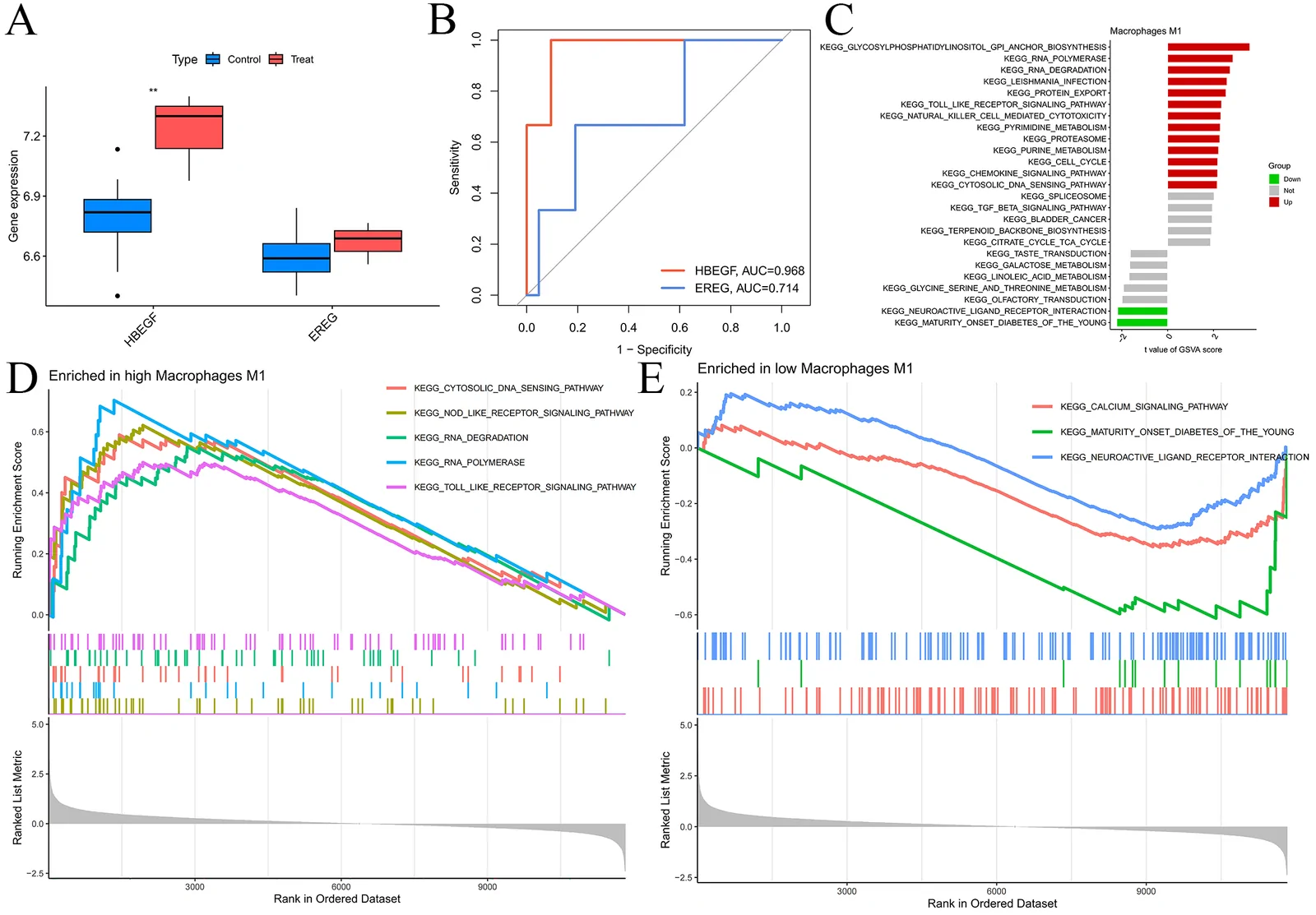
External validation of hub gene expression and related pathways based on independent GEO dataset GSE80496. **(A)** Expression levels of the two hub genes in the validation cohort. *p < 0.05; **p < 0.01; ***p < 0.001. **(B)** ROC curve of *HBEGF* in the validation cohort. **(C)** Gene Set Variation Analysis (GSVA) comparing the high- and low–M1 macrophage groups. **(D)** Gene Set Enrichment Analysis (GSEA) of the high–M1 macrophage group. **(E)** GSEA of the low–M1 macrophage group.

GSVA (**Figure 8C**) indicated that the M1 macrophage high-expression group was predominantly enriched in glycosylphosphatidylinositol (GPI)-anchor biosynthesis, RNA polymerase, and RNA degradation pathways. In contrast, the M1 macrophage low-expression group was mainly enriched in maturity-onset diabetes of the young and neuroactive ligand–receptor interaction pathways. GSEA further indicated that the M1 macrophage high-expression group (**Figure 8D**) was predominantly enriched in the cytosolic DNA-sensing pathway, NOD-like receptor signaling pathway, RNA degradation, RNA polymerase, and Toll-like receptor signaling pathway, whereas the M1 macrophage low-expression group (**Figure 8E**) was primarily enriched in the calcium signaling pathway, maturity-onset diabetes of the young, and neuroactive ligand–receptor interaction pathways.

### 3.8 Experimental validation of hub gene expression

RT-qPCR was conducted to measure the mRNA expression levels of the two hub genes in peripheral blood samples obtained from the control group and individuals with meningitis. The bar plot results (**Figure 9A**) indicated that, compared with the control group, the expression levels of *HBEGF* and *EREG* were both significantly upregulated in the peripheral blood obtained from individuals with meningitis (*p* < 0.001), consistent with the sequencing results derived from public datasets. To further validate protein expression levels of the two hub genes, WB was performed in 9 samples per group (one sample from each group was excluded from densitometric analysis due to poor ACTB band quality). The bar plot results (**Figure 9B**) indicated that, compared with the control group, the protein expression levels of *HBEGF* and *EREG* were both increased in the peripheral blood obtained from individuals with meningitis, with statistically significant differences between groups (*p* < 0.05). Densitometric analysis (**Figure 9C**) indicated that band intensities corresponding to *HBEGF* and *EREG* were markedly increased in samples obtained from individuals with meningitis.

**Figure 9:**
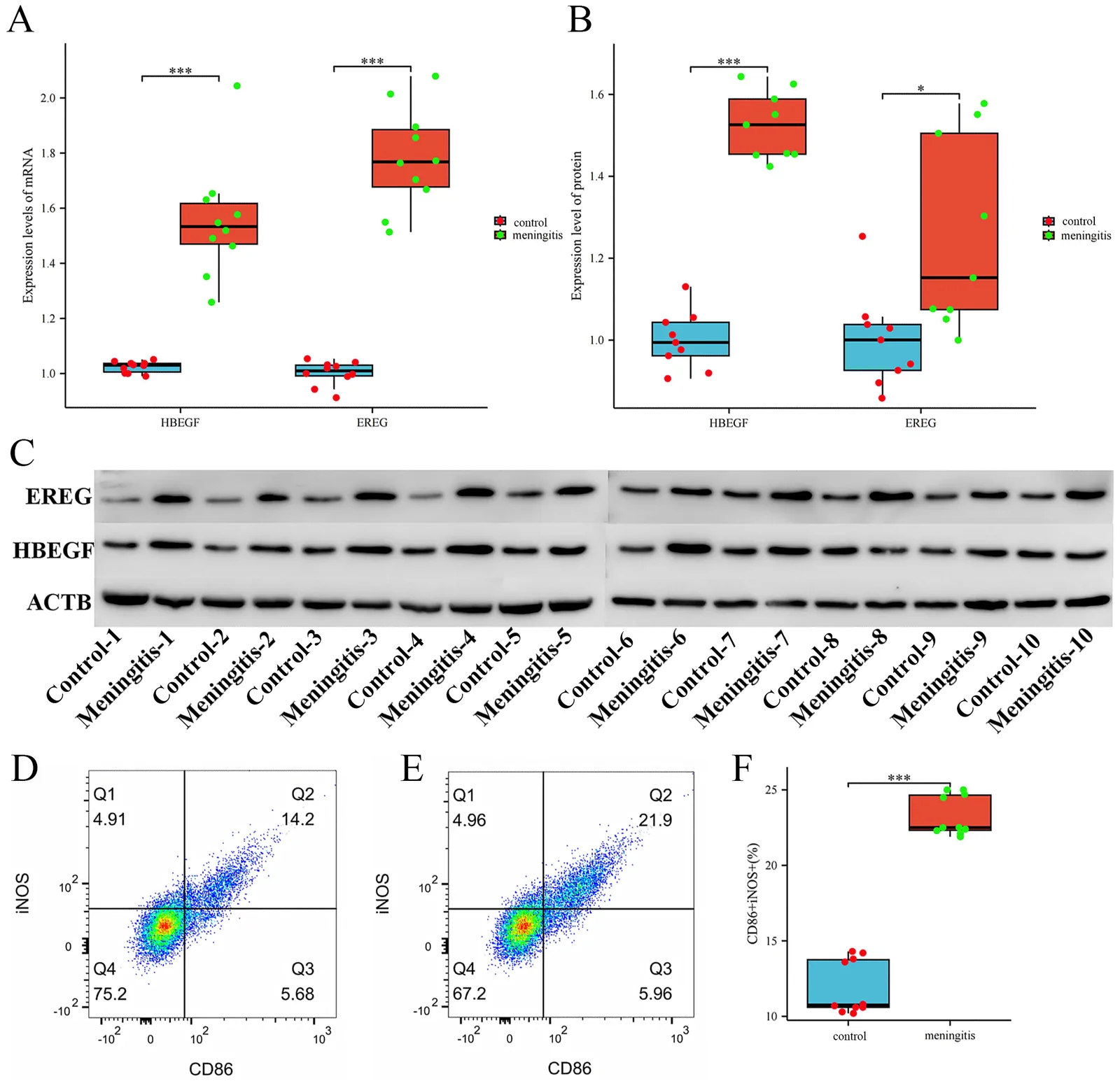
Experimental validation of HBEGF and EREG expression and M1 macrophage proportion in clinical peripheral blood samples via RT-qPCR, Western blotting and flow cytometry. **(A)** mRNA expression levels validated by reverse transcription–quantitative polymerase chain reaction (RT-qPCR). **(B)** Densitometric quantitative statistics of HBEGF and EREG relative protein levels normalized to ACTB, with intergroup Student’s t-test results displayed. **(C)** Raw western blotting images showing target protein bands (HBEGF, EREG) and housekeeping protein ACTB in meningitis patients and individuals from the non-meningitis neurological disease control group. **(D)** Representative flow cytometry plot showing the proportion of M1 macrophages in peripheral blood from individuals from the nonmeningitis neurological disease control group. **(E)** Representative flow cytometry plot showing the proportion of M1 macrophages in peripheral blood from individuals with meningitis. **(F)** Bar plot comparing the proportion of M1 macrophages in peripheral blood between the individuals from the nonmeningitis neurological disease control group and meningitis groups. For WB densitometric quantification and statistical analysis (panels **(B, C)**), 9 samples per group were included. All original WB bands from the total 10 sample pairs are shown in panel **(C)**, while one sample from each group was excluded from densitometry due to relatively low ACTB signal and poor band quality. *p < 0.05; **p < 0.01; ***p < 0.001.

### 3.9 Proportion of M1 macrophages in peripheral blood

The proportion of M1 macrophages in peripheral blood was quantified in 20 samples, as presented in **Supplementary Table 3** and illustrated by representative flow cytometry dot plots (**Figures 9D, E**). Comparative analysis of M1 macrophage proportions between the two groups (**Figure 9F**) indicated that individuals with meningitis exhibited a significantly higher proportion of M1 macrophages in peripheral blood compared with the control group (*p* < 0.001).

## 4 Discussion

Meningitis is an acute or chronic inflammatory disorder of the meninges caused by pathogenic microorganisms or by physical and chemical factors (Aksamit and Berkowitz, 2021; Hasbun, 2022; Ivaska et al., 2024). The underlying pathogenic mechanisms remain incompletely understood. Individuals with meningitis frequently present with severe symptoms at the time of diagnosis, require hospitalization, and often experience poor prognosis despite treatment. Consequently, early detection and accurate diagnosis have become key clinical priorities (Abe et al., 2021; Yekani and Memar, 2023). Prior studies have demonstrated that biomarkers such as interleukin-6 (IL-6), procalcitonin, C-reactive protein (CRP), and ferritin may serve as early diagnostic biomarkers for bacterial meningitis (Groeneveld et al., 2025). At the gene expression level, Jiang et al. reported that *RNF144B* and *FYN* exhibited opposing expression patterns in peripheral blood across different stages of bacterial meningitis, indicating their potential utility as biomarkers for disease diagnosis and monitoring (Jiang et al., 2025).

With advances in bioinformatics, machine learning algorithms and WGCNA have become well established, and their applications in biomarker prediction and therapeutic target identification have expanded considerably. In the present study, integrated analysis of multiple GEO datasets identified 292 DEGs associated with meningitis, including 97 downregulated and 195 upregulated genes (Liu et al., 2017; Sarker, 2021). Immune cell infiltration analysis further indicated that M1 macrophages and activated dendritic cells were significantly increased in samples obtained from individuals with meningitis. By combining WGCNA, PPI network analysis, and machine learning approaches, *HBEGF* and *EREG* were identified as two hub genes associated with meningitis. Experimental validation using RT-qPCR, WB, and flow cytometry subsequently confirmed that both genes were significantly upregulated in peripheral blood samples obtained from individuals with meningitis, accompanied by an increased proportion of M1 macrophages.

*HBEGF* is a member of the epidermal growth factor (EGF) family. It is synthesized as a transmembrane precursor (proHB-EGF) and can be cleaved by metalloproteinases of the ADAM metalloproteinases to generate a soluble form (sHB-EGF) (Dreux et al., 2006; Dao et al., 2018). By binding to receptors such as EGFR (HER1) and ERBB4, *HBEGF* activates downstream signaling pathways including MAPK and PI3K signaling pathways, thereby regulating cell proliferation, migration, differentiation, and survival, and contributing to physiological processes such as wound repair and tissue development (Higashiyama et al., 1992; Dreux et al., 2006; Dao et al., 2018).

In tumors, HBEGF binds to EGFR on the tumor cell surface in a paracrine manner, leading to activation of downstream MAPK and PI3K/AKT signaling pathways and induction of epithelial–mesenchymal transition. This process is characterized by downregulation of E-cadherin and upregulation of mesenchymal markers such as Slug and ZEB1/2, thereby promoting tumor cell migration and invasion (Raaschou-Nielsen et al., 2013; Hamra et al., 2014; Guan et al., 2016).

Shin et al. demonstrated that, in the context of Ink4a/Arf and Pten deficiency, *Hbegf* was sufficient to initiate glioblastoma in mice, and that these tumors exhibited molecular characteristics similar to the classical glioblastoma subtype observed in individuals with the disease. *In vivo* inhibition of *Hbegf* resulted in tumor regression and significantly improved survival (Shin et al., 2017). Kuo et al. reported that *HBEGF*-positive macrophages promoted fibroblast invasiveness through an EGFR-dependent mechanism, and that intercellular signaling within the inflammatory environment altered cellular phenotypes and contributed to fibroblast-mediated joint destruction (Kuo et al., 2019).

Although direct mechanistic evidence in meningitis remains limited, this study demonstrated increased expression of *HBEGF* in peripheral samples obtained from individuals with meningitis, accompanied by a significant increase in M1 macrophage abundance. Compared with the classic diagnostic biomarkers of meningitis such as IL-6, procalcitonin (PCT), CRP and S100B, HBEGF and EREG identified in this study have better diagnostic performance for meningitis, with the combined AUC reaching 0.922. In addition, unlike the non-specific inflammatory indicators such as CRP and PCT, HBEGF and EREG are closely related to the polarization of M1 macrophages, which can reflect the underlying immune regulatory mechanism of meningitis, and have higher mechanism specificity. However, the diagnostic value of these two genes in distinguishing different etiological types of meningitis needs to be further verified. These findings indicate that *HBEGF* may contribute to M1 macrophage polarization through activation of the EGFR/ErbB signaling pathway, thereby amplifying inflammatory responses during meningitis.

*EREG* encodes epiregulin, a soluble secreted protein belonging to the EGF family (Kohsaka et al., 2014). In inflammatory settings, epiregulin has been implicated in tissue repair, wound healing, and oocyte maturation through regulation of angiogenesis, vascular remodeling, and cellular proliferation (Kohsaka et al., 2014). Ran et al. reported that, under both normal and inflammatory conditions, targeted knockdown of *EREG* activated the p38 MAPK and Erk pathways in dental pulp stem cells (DPSCs), which significantly enhanced their osteogenic and odontogenic differentiation potential by increasing phosphorylation levels of p38 MAPK and Erk (Ran et al., 2021).

In tumors, epiregulin can sustain prosurvival and proliferative signaling through autocrine or paracrine mechanisms, thereby promoting tumor initiation, progression, invasion, and metastasis and contributing to resistance to chemotherapy (Cheng et al., 2021). A study on cervical cancer demonstrated that *EREG* promotes the proliferation, migration, invasion, and tube formation of human umbilical vein endothelial cells (HUVECs) through interactions with receptors such as EGFR and ERBB4 and has been reported as an independent prognostic biomarker associated with tumor prognosis and immunotherapy response across multiple cancer types (Yang et al., 2023).

In this study, EREG expression was elevated in individuals with meningitis and was enriched in inflammation-related pathways, including the ErbB, PI3K/Akt, and MAPK signaling pathways, indicating that increased EREG expression may contribute to the initiation and progression of inflammation through activation of these downstream pathways.

The present study also demonstrated a significant increase in the proportion of M1 macrophages in peripheral blood samples obtained from individuals with meningitis, consistent with findings reported by Suwanruengsri et al. in Japanese Black cattle (Suwanruengsri et al., 2022). During inflammatory responses, classically activated (M1) macrophages release proinflammatory cytokines, including tumor necrosis factor-alpha (TNF-α), interleukin-1 beta, IL-12, and IL-23, in response to inflammatory stimuli. Persistent activation of the M1 macrophage phenotype may result in tissue damage (Shapouri-Moghaddam et al., 2018; Luo et al., 2024). Prior studies have also demonstrated that M1 macrophages in bacterial meningitis can contribute to both pathogen elimination and the exacerbation of tissue injury (Hansakon et al., 2020). Functional enrichment analysis in the present study indicated that the high–M1 macrophage group was enriched in innate immune activation pathways, including the Toll-like receptor and NOD-like receptor signaling pathways, further supporting a role for M1 macrophage–mediated immune activation in meningitis.

Immune cell correlation analysis indicated a positive association between M1 macrophages and γδ T cells, suggesting potential immunological crosstalk between these cell populations. M1 macrophages represent a macrophage phenotype classically activated by interferon-γ (IFN-γ) and lipopolysaccharide and exhibit potent proinflammatory and microbicidal functions (Yunna et al., 2020). γδ T cells constitute a subset of innate-like T lymphocytes characterized by expression of the γδ T-cell receptor, which enables rapid recognition of a broad range of antigens without major histocompatibility complex restriction and supports cytotoxic and immune surveillance functions (Ribot et al., 2021).

Notably, γδ T cells have been reported to represent an important source of IFN-γ following spinal cord injury, and IFN-γ signaling can promote macrophage polarization toward the proinflammatory M1 phenotype, thereby increasing the secretion of inflammatory cytokines such as TNF-α and exacerbating tissue injury (Wo et al., 2020). In meningitis, *HBEGF* and *EREG* may function as key molecular links connecting inflammatory signaling, macrophage activation, and immune cell recruitment, and their expression levels may serve as adjunctive diagnostic biomarkers for meningitis.

Although HBEGF and EREG were identified as M1 macrophage-related meningitis biomarkers, several limitations exist. The clinical validation cohort in the present study contained 10 meningitis patients and 10 individuals from the non-meningitis neurological disease control group. Simultaneous performance of western blotting, RT-qPCR and flow cytometry requires sufficient volume and high-quality peripheral blood specimens; eligible meningitis subjects meeting strict inclusion criteria were difficult to recruit during the study period, leading to this relatively small exploratory sample size. Our clinical validation involved a small single-center cohort without multi-etiology stratification, restricting result generalizability. We analyzed bulk transcriptomes via CIBERSORT without single-cell data, and all validations used peripheral blood rather than cerebrospinal fluid. No pathogen subgroup labels in datasets prevented subtype comparison or adjustment of infection-related confounders; thus the two markers only reflect pan-meningitis inflammation and cannot differentiate bacterial/viral cases. Moreover, only correlative evidence was obtained, lacking cell and animal experiments to prove causal regulation of M1 polarization. We strengthened result reliability via cross-validation and external datasets, and further multi-center, functional studies are warranted. In addition, most meningitis patients received empirical antibiotic treatment before blood sampling. Antibiotic intervention may alter peripheral inflammatory gene and protein expression, which could constitute a confounding factor in our observational analysis.

## 5 Conclusion

Through integrated multi-omics analysis, *HBEGF* and *EREG* were identified as macrophage-associated hub genes in meningitis and were shown to be closely associated with M1 macrophage infiltration. These genes may serve as potential diagnostic biomarkers for meningitis and provide clues for exploring future therapeutic targets.

## Data Availability

The publicly available datasets analyzed in this study can be obtained from the Gene Expression Omnibus (GEO) repository under accession numbers GSE11199, GSE29507, GSE82166, GSE82316, and GSE80496. The experimental data generated in this study are available from the corresponding authors upon reasonable request.
