# Supplementary material for: Integrated multi-omics analysis and experimental validation identify hub genes with diagnostic value in meningitis
Source: Front Cell Infect Microbiol. 2026 Sep 16;16:1839548. doi: 10.3389/fcimb.2026.1839548 (PMC13623609; doi:10.3389/fcimb.2026.1839548)
Supplement: Supplementary file 1 [file Table1.docx]

**Supplementary Table 1** Descriptive statistics of the GEO datasets

| Database | Platform | Dataset | Sample | | Species |
| --- | --- | --- | --- | --- | --- |
|  |  |  | Meningitis | Control |  |
| GEO | GPL570 | GSE11199 | 4 | 4 | Homo sapiens |
| GEO | GPL4133 | GSE29507 | 5 | 4 | Homo sapiens |
| GEO | GPL6244 | GSE82166 | 5 | 5 | Homo sapiens |
| GEO | GPL6244 | GSE82316 | 4 | 2 | Homo sapiens |
| GEO | GPL6883 | GSE80496 | 3 | 21 | Homo sapiens |
