# Supplementary material for: Integrated multi-omics analysis and experimental validation identify hub genes with diagnostic value in meningitis
Source: Front Cell Infect Microbiol. 2026 Sep 16;16:1839548. doi: 10.3389/fcimb.2026.1839548 (PMC13623609; doi:10.3389/fcimb.2026.1839548)
Supplement: Supplementary file 2 [file Table2.docx]

**Supplementary Table 2** The primer sequences for RT-qPCR.

| Gene | Primer sequence |
| --- | --- |
| HBEGF | F: 5'-TTCTGGCTGCAGTTCTCTCG-3' |
|  | R: 5'-AAGTCACGGACTTTCCGGTC-3' |
| EREG | F: 5'-ATCACAGTCGTCGGTTCCAC-3' |
|  | R: 5'-AGGCACACTGTTATCCCTGC-3' |
