# Supplementary material for: Integrated multi-omics analysis and experimental validation identify hub genes with diagnostic value in meningitis
Source: Front Cell Infect Microbiol. 2026 Sep 16;16:1839548. doi: 10.3389/fcimb.2026.1839548 (PMC13623609; doi:10.3389/fcimb.2026.1839548)
Supplement: Supplementary file 3 [file Table3.docx]

**Supplementary Table 3** Proportion of M1 macrophages in peripheral blood samples (%)

| No. | 1 | 2 | 3 | 4 | 5 | 6 | 7 | 8 | 9 | 10 |
| --- | --- | --- | --- | --- | --- | --- | --- | --- | --- | --- |
| Normal | 14.2 | 13.8 | 10.8 | 10.7 | 10.6 | 10.2 | 14.3 | 13.6 | 10.3 | 10.6 |
| Meningitis | 21.9 | 24.7 | 22.3 | 22.5 | 22.4 | 22.1 | 24.5 | 22.5 | 25.0 | 25.0 |
