# Supplementary material for: Integrated multi-omics analysis and experimental validation identify hub genes with diagnostic value in meningitis
Source: Front Cell Infect Microbiol. 2026 Sep 16;16:1839548. doi: 10.3389/fcimb.2026.1839548 (PMC13623609; doi:10.3389/fcimb.2026.1839548)
Supplement: Supplementary file 4 [file Table4.docx]

**Supplementary Table4** Baseline clinical characteristics of study participants

*Abbreviations: M, meningitis group; C, non-meningitis neurological disease control group; NA, not available.*

| **Sample ID** | **Group** | **Age (years)** | **Sex** | **Primary diagnosis** | **Comorbidities / Complications** | **Sampling time point** | **Prior empirical antibiotic therapy** |
| --- | --- | --- | --- | --- | --- | --- | --- |
| M1 | Meningitis | 56 | Male | Meningitis / Intracranial infection | Right sphenoid ridge meningioma; Right frontotemporal large-area cerebral infarction | NA | Yes |
| M2 | Meningitis | 43 | Male | Meningitis / Intracranial infection | Thalamic hemorrhage with obstructive hydrocephalus | NA | Yes |
| M3 | Meningitis | 64 | Male | Meningitis / Intracranial infection | Brain abscess; Hydrocephalus | NA | Yes |
| M4 | Meningitis | 53 | Female | Fungal meningitis / Intracranial infection | Septic shock; Acute heart failure | NA | Yes |
| M5 | Meningitis | 59 | Female | Meningitis / Intracranial infection | Mental disorder due to intracranial infection | NA | Yes |
| M6 | Meningitis | 41 | Male | Meningitis / Intracranial infection | Pulmonary infection; Acute respiratory failure | NA | Yes |
| M7 | Meningitis | 15 | Female | Meningitis / Intracranial infection | Cerebellar vermis intracranial space-occupying lesion | NA | Yes |
| M8 | Meningitis | 51 | Male | Meningitis / Intracranial infection | Right basal ganglia hemorrhage | NA | Yes |
| M9 | Meningitis | 71 | Female | Meningitis / Intracranial infection | History of cerebral hemorrhage | NA | Yes |
| M10 | Meningitis | 31 | Male | Meningitis / Intracranial infection | Hydrocephalus; Frontal bone fracture | NA | Yes |
| C1 | Control | 60 | Female | Cerebral infarction (recovery stage) | Posterior communicating artery aneurysm (post clipping and wrapping) | NA | No |
| C2 | Control | 51 | Male | Left temporal dural arteriovenous fistula with subarachnoid hemorrhage | Status post hematoma evacuation | NA | No |
| C3 | Control | 49 | Male | Left frontal and occipital intracranial space-occupying lesion | Right frontal cerebral infarction; Status post endoscopic lesion resection | NA | No |
| C4 | Control | 73 | Female | Cerebral infarction | Pulmonary infection; Status post ventricular drainage | NA | No |
| C5 | Control | 51 | Male | Right basal ganglia hemorrhage | Left limb weakness; Status post endoscopic hematoma drainage | NA | No |
| C6 | Control | 59 | Male | Right temporal glioma | Status post endoscopic skull base lesion resection | NA | No |
| C7 | Control | 15 | Male | Cerebrospinal fluid leak | Status post meningocele repair and CSF leak repair | NA | No |
| C8 | Control | 37 | Female | Cerebral arteriovenous malformation | Left thalamic hemorrhage; Status post embolization | NA | No |
| C9 | Control | 51 | Male | Left cerebellopontine angle intracranial space-occupying lesion | Brainstem and prepontine cistern lesion; Status post endoscopic skull base lesion resection | NA | No |
| C10 | Control | 67 | Female | Acute cerebrovascular disease | Cerebral infarction due to major artery occlusion with left hemiplegia; Status post decompressive craniectomy | NA | No |

**Notes:** All participants were enrolled from a single‑center cohort. The meningitis group (M1–M10) comprised patients with clinically diagnosed meningitis/intracranial infection. Most patients in the meningitis group received empirical antibiotic therapy prior to sample collection; peripheral blood samples were obtained at the earliest time‑point after the clinical diagnosis of meningitis was established. The control group (C1–C10) comprised hospitalized patients with non‑meningitis neurological diseases, not healthy volunteers, who did not receive anti‑meningitis targeted antibiotic treatment. Peripheral blood samples were collected for RT‑qPCR, western blot, and flow cytometry assays. Bed numbers and hospitalization IDs were removed for patient de‑identification. Precise hours from disease onset to sampling were not consistently available in retrospective clinical records and are marked as NA.
